# Candidate Gene Selection for Cytoplasmic Male Sterility in Pepper (*Capsicum annuum* L.) through Whole Mitochondrial Genome Sequencing

**DOI:** 10.3390/ijms20030578

**Published:** 2019-01-29

**Authors:** Peng Wang, Qiaohua Lu, Yixin Ai, Yihao Wang, Tiantian Li, Lang Wu, Jinqiu Liu, Qing Cheng, Liang Sun, Huolin Shen

**Affiliations:** 1 Beijing Key Laboratory of Growth and Developmental Regulation for Protected Vegetable Crops, China Agricultural University, Beijing 100193, China; wp737@outlook.com (P.W.); lqh12261842@163.com (Q.L.); aiyixin0129@126.com (Y.A.); yhwang0906@126.com (Y.W.); ltt737737@163.com (T.L.); 15291832633@163.com (L.W.); 18811797502@163.com (J.L.); chengqing2013@126.com (Q.C.); 2 Department of Vegetable Science, College of Horticulture, China Agricultural University, No. 2 Yuanmingyuan Xi Lu, Haidian District, Beijing 100193, China

**Keywords:** cytoplasmic male sterility (CMS), pepper (*Capsicum annuum* L.), mitochondria, CMS-associated gene

## Abstract

Cytoplasmic male sterility (CMS), which is controlled by mitochondrial genes, is an important trait for commercial hybrid seed production. So far, genes controlling this trait are still not clear in pepper. In this study, complete mitochondrial genomes were sequenced and assembled for the CMS line 138A and its maintainer line 138B. The genome size of 138A is 504,210 bp, which is 8618 bp shorter than that of 138B. Meanwhile, more than 214 and 215 open reading frames longer than 100 amino acids (aas) were identified in 138A and 138B, respectively. Mitochondrial genome structure of 138A was quite different from that of 138B, indicating the existence of recombination and rearrangement events. Based on the mitochondrial genome sequence and structure variations, mitochondrion of 138A and FS4401, a Korean origin CMS line, may have inherited from a common female ancestor, but their CMS traits did originate separately. Candidate gene selection was performed according to the published characteristics of the CMS genes, including the presence SNPs and InDels, located in unique regions, their chimeric structure, co-transcription, and transmembrane domain. A total of 35 ORFs were considered as potential candidate genes and 14 of these were selected, with *orf300a* and *0rf314a* as strong candidates. A new marker, orf300a, was developed which did co-segregate with the CMS trait.

## 1. Introduction

Heterosis plays an important role in crop production. Hybrid crops produce 15–50% higher yields than the parent lines [1]. Therefore, heterosis has been widely used in the production of many cereal and horticultural crops, such as rice, maize, rapeseed, sorghum, sunflower, cucumber, watermelon, tomato as well as pepper [2]. However, one of the factors that limits the application of heterosis is emasculation, which is not only time-consuming and costly, but also unaffordable for many self-pollinating crops. Fortunately, the use of a male sterile line, which does not need emasculation during hybrid production, is a good solution for overcoming this problem. Since it was first successfully utilized in the production of hybrid corn (the maize CMS-T (Texas) system), the male-sterile-line-based hybrid technique has been applied in many other crops [3]. 

Cytoplasmic male sterility (CMS) is caused by sterility genes in the cytoplasm which lead to the abnormal stamen and incompatible pollen. Cytoplasmic male sterility can be restored by the restorer of fertility (*Rf*) gene in the nucleus. In contrast to genic male sterility (GMS), CMS is more widely used because the male-sterility trait can be maintained efficiently. Cytoplasmic male sterility is often found in higher plants and features maternal inheritance, pollen abortion, and normal pistil. It is often caused by the expression of a novel open reading frame (ORF) which is located on the mitochondrial genome [4,5]. Mitochondrial genomes of higher plants have many unique characteristics that are different from those of animals and fungi [6]. The most notable feature of the mitochondrial genome of higher plants is its genome size, which range from 200 to 2400 kb [7]. The mitochondrial genome of *Brassica hirta* is 208 kb [8], pepper 512 kb [9], and *Silene* 11.3 MB [10]. A large genome size of the plant mitochondrial genome often results in a large number of non-coding sequences, including gene spacing, repeat sequences, and introns. In addition, higher plant mitochondrial genomes usually show higher recombination and rearrangement rates, which could be attributed to the presence of repetitive sequences, which are considered to be one of the main drivers of the mitochondrial genome rearrangement [11,12,13,14,15]. Active recombination and rearrangement in plant mitochondrial DNA often lead to the generation of genes with altered ORFs, which are often manifested as aberrant chimeric structures or co-transcribed with genuine mitochondrial genes [5]. The expression of these aberrant chimeric genes at the RNA or protein level might affect the normal function of an inserted or co-transcribed gene and further affects the normal function of the mitochondria [11]. Those aberrant ORFs in higher plants are often considered as one of the main reasons for CMS.

Since the discovery of male sterility by the German botanist Joseph Gottlieb Kolreuter in 1763 [16], plants with male sterility have been identified in 43 families, 162 genera, and approximately 617 species [17]. More than 28 CMS-related genes have been identified in 13 crop species [3]. These genes often show chimeric structures with the mitochondrial genes present in the normal mitochondria, and encode proteins with transmembrane domains. For instance, the *orf79* and its variant *orfH79* control the CMS-BT and CMS-HL in rice, respectively. Both of them encode proteins with chimeric structures, of which the N terminus sequences are similar to that of the *cox1* gene and the remaining regions could not be aligned to any known proteins [18]. In radish, *orf463* controls the CMS-DCGMS, of which the 5′ end 128 bp region can be aligned to *cox1* gene and the remaining region could not match any known genes [19]. Meanwhile, *orf463* are predicted to contain 12 potential transmembrane domains. Although chimeric structure is one of the most notable features of the CMS genes, it is not an indispensable characteristic for the CMS gene. For example, in rice, *orf352* has been confirmed to cause the CMS trait, which does not feature the chimeric structure, but co-transcribes with the ribosomal protein gene *rpl5* [20]. 

The CMS sterility caused by novel mitochondrial ORFs can be restored by the *Rf* genes in the nucleus. Until now, more than ten *Rf* genes have been identified, such as *Rf2* in maize [21,22], *Rf17* in rice [23,24], *Rf-PPR592* in *Petunia* [25], and *PPR6* in pepper [26]. Most of the *Rf* genes are pentatricopeptide repeat (PPR) genes, which encode a group of RNA-binding proteins that often act in the organellar posttranscriptional mRNA processes, such as editing, splicing, cleavage, degradation, and translation [27,28].

Pepper is an important vegetable crop and has been widely grown all over the world. Cytoplasmic male sterility has been widely used in hybrid pepper seed production. A number of studies have been conducted to identify candidate genes controlling CMS. So far, two candidate genes have been reported by a Korean group via restriction fragment length polymorphism (RFLP) analyses and RT-PCR [29,30,31,32]. One candidate, the *atp6* gene, encoding subunit of the mitochondrial ATP synthase complex, has been identified to be a gene duplication in the mitochondrial genome. The first copy of the *atp6* gene, *atp6-1*, shared the same sequence in both of the male-sterile (Milyang-CMS) and maintainer lines (Milyang-N). However, in regard to the second copy of the *atp6* gene, *atp6-2*, there is a deletion at the 3′ end of the male-sterile allele (*ψatp6-2*), which is considered to be the reason for the male-sterile trait [29,31]. Another candidate, identified in Milyang-CMS and 417/A, is the *orf456*/507 gene, which is called *orf456* by Kim et al. [32], and *orf507* by Gulyas [33]. The gene *orf507* harbors a single nucleotide deletion at the +449 position which increases the ORF from 456 bp to 507 bp [32,33]. Recently, Jo et al. [9] published the complete mitochondrial genome sequence of the pepper CMS line FS4401 and the fertile line Jeju. *orf507* and *ψatp6-2* were only identified in the mitochondrial genome of FS4401, further indicating they may be the candidates controlling CMS.

In this study, we sequenced and assembled the mitochondrial genomes of the CMS line 138A as well as its maintainer line 138B. A mitochondrial genome wide pairwise comparison was also conducted among the two Korean (FS4401 and Jeju) and two Chinese (138A and 138B) materials. Based on the genome sequence variation as well as the expression patterns, several candidate mitochondrial genes responsible for the male sterility in 138A were predicted.

## 2. Results

### 2.1. Mitochondrial Genome Sequence of CMS Line 138A and Its Maintainer Line 138B

Mitochondrial genomes of CMS line 138A and its maintainer line 138B were sequenced via Illumina PE and PacBio techniques. For the Illumina PE sequencing, 5429 Mb and 6837 Mb clean data were obtained from line 138A and 138B, respectively. The Q30 values for 138A and 138B were 94.73% and 95.07%, respectively. In regard to the PacBio sequencing, 98.36 Mb filtered subreads with N50 = 10,278 bp and 101.83 Mb filtered subreads with N50 = 10,357 bp were generated from line 138A and B, respectively. Through de novo assembling, mitochondrial genomes of 138A and 138B were obtained, of which the first one was 504,210 bp and second one was 512,828 bp. The GC contents of the mitochondrial genomes of 138A and 138B were 44.55% and 44.51%, respectively. The complete mitochondrial genome sequences of line 138A and 138B can be obtained in the GenBank nucleotide sequence database (https://www.ncbi.nlm.nih.gov/genbank/) under the accession numbers of MK225636 and MK225637, respectively.

### 2.2. ORF Identification and Gene Annotation

**Figure 1:**
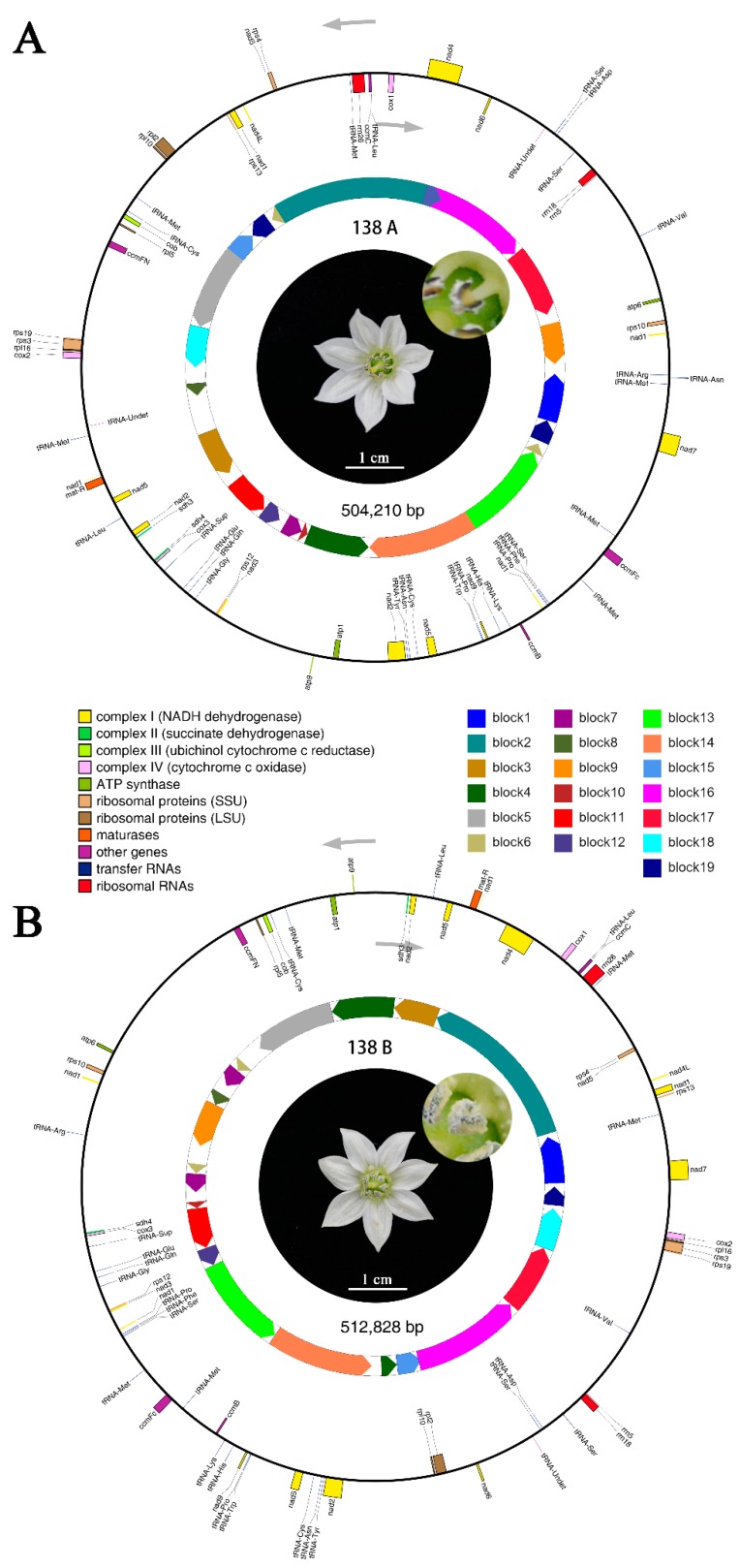
Mitochondrial genome maps of sterile line 138A and maintainer line 138B. (**A**) Mitochondrial genome map of 138A. (**B**) mitochondrial genome map of 138B. Genes with the names inside the circle are transcribed clockwise. Genes with names outside the circle are transcribed counterclockwise. The colors of the genes denote the functions of the gene products. Syntenic sequence blocks between genomes are depicted on the inner circles to separate blocks in different directions. In the center of the maps are the sterile flower of 138A and the normal fertile flower of 138B, respectively.

The ORF encoding proteins that were equal to or larger than 100 aas were identified from the assembled mitochondrial genomes. In 138A, 214 ORFs (including 33 known genes) and 34 non-coding RNAs (ncRNAs) were identified, and meanwhile 215 ORFs (including 33 known genes) and 31 ncRNAs were recognized in 138B (Supplementary Materials Table S1). Among those ORFs, 27 and 26 unique ORFs were identified in 138A and 138B, respectively (Supplementary Materials Tables S2 and S4). As to the known genes in 138A and 138B mitochondrial genomes, there was one-to-one correspondence with each other and encoded ribosomal protein large subunits RPL2, RPL5, RPL10, RPL16, RPS3, RPS4, RPS10, RPS12, RPS13, and RPS19; succinic acid dehydrogenase subunits SDH3 and SDH4; ATP synthase ATP1, ATP6, and ATP9; cytochrome oxidase subunits COX1, COX2, and COX3; cytochrome b *COB*; NADH dehydrogenase subunits NAD1, NAD2, NAD3, NAD4, NAD4L, NAD5, NAD6, NAD7, and NAD9; cytochrome C synthesis related proteins CCMB, CCMC, CCMFC, and CCMFN; mature enzymes MATR. Finally, by combining with the mitochondrial sequences and ORFs identification results, mitochondrial genome maps of 138A and 138B were constructed (Figure 1). 

### 2.3. Comparative Analysis of the Mitochondrial Genomes

#### 2.3.1. Syntenic Sequence Analysis of the Mitochondrial Genomes

**Figure 2:**
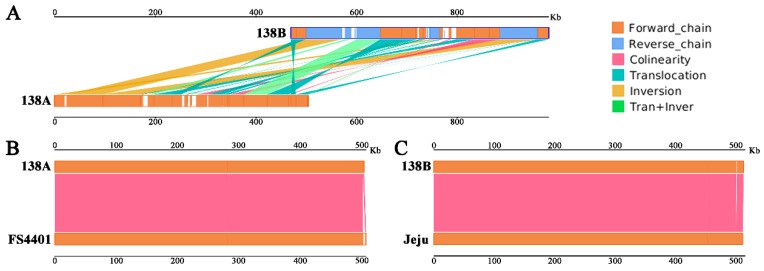
Mitochondrial genome syntenic sequence analysis. (**A**) Syntenic sequence analysis of 138A and 138B; (**B**) syntenic sequence analysis of 138A and FS4401; (**C**) syntenic sequence analysis of 138B and Jeju. The upper and lower bars in each figure represented the mitochondrial genomes. Dark-orange and blue regions in each bar represented the forward and reverse direction of the aligned genome, respectively. White regions in each bar represent the sequences that could not be aligned to the other genome. Lines between the two bars indicated the syntenic types and locations: magenta, blue-green, dark yellow, and light-green represent for collinear, translocation, inversion, and tran + inver, respectively.

Comparative analysis was conducted between the of Korean CMS line FS4401 and its fertile line Jeju and CMS line 138A and maintainer line 138B. In terms of the genome size, 138A was 3242 bp smaller than FS4401 (507,452 bp), while 138B was 1298 bp larger than Jeju (511,530 bp). In regard to the known functional gene annotation, compared with FS4401 and Jeju, both 138A and 138B lacked three genes, including *atp4*, *atp8*, and *mttB*. A total of 19 syntenic sequence blocks were identified between 138A and 138B (Figure 1), which account for 91.68% and 90.24% of the mitochondrial genome sequences, respectively. A total of 23 unique regions ranging from 19 bp to 10,469 bp were identified between 138A and 138B, of which 11 unique regions were found in 138A genome, and the other 12 unique regions were found in 138B genome (Supplementary Material Tables S3 and S5). In regard to the whole genome structure, there was an extensive recombination and rearrangement between the two mitochondrial genomes (Figure 2A). In contrast, a very high degree of collinearity, reaching more than 99%, was observed between the CMS lines and fertility lines (Figure 2B,C).

#### 2.3.2. Genome Structural Variation between 138A and 138B

**Figure 3:**
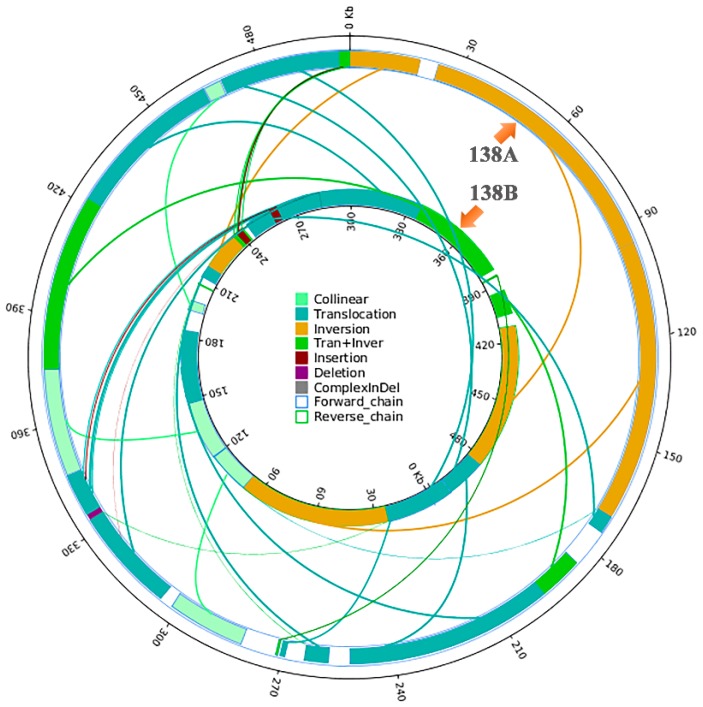
Structural variation map of mitochondrial genomes of 138A and 138B. The inner circle is the 138B genome, and the outer circle is the 138A genome. Collinear: the same linear region; Translocation: the area of translocation; Inversion: the area of inversion; Tran + Inver: the area of translocation and inversion; Insertion: the insertion region with a length greater than or equal to 50 bp; Deletion: the deletion region with length greater than or equal to 50 bp; Complex InDel: an area that cannot be compared, but corresponds to a position; Forward_chain: forward chain of the genome sequence, at which time the gene coordinates increase in a clockwise direction; Reverse_chain: the reverse chain of the genome sequence in which the genetic coordinates increase counterclockwise.

For further clarifying the difference between the two mitochondrial genomes, genome structural variations were investigated between 138A and 138B using 138B as reference. As shown in Figure 3, 13 ectopic, three inversion and eight ectopic + inversion regions were recognized between the two genomes (Figure 3). Meanwhile, two insertions and one deletion which were longer than 50 bp were also identified in the syntenic regions.

#### 2.3.3. SNP and InDel Detection

In order to identify sequence variations in the known genes as well as the ORFs between 138A and 138B mitochondrial sequences, SNPs and InDels were detected between the two mitochondrial genomes. As shown in Tables S6–S8, a total of 112 SNPs and 14 InDels were identified between the two mitochondrial genomes. Among those mutations, only one SNP was identified in the known mitochondrial gene, *mat-R*; however, this SNP does not lead to non-synonymous mutations, suggesting it may not be the reason for the CMS. In another aspect, although seven SNPs which led to non-synonymous mutations were identified in six common ORFs shared by both 138A and 138B, no chimeric structure was identified in those ORFs (Supplementary Materials Table S11). In regard to the InDels, two were observed in two common genes shared by 138A and 138B; however, none of them resulted in the frame-shift mutation (Supplementary Materials Table S11). 

### 2.4. Selection of Candidate Genes Controlling the Cytoplasmic Male Sterility

#### 2.4.1. Identification of Novel ORFs in the Unique Regions of 138A

Based on previous studies, novel ORFs generated from the recombination and rearrangement of the mitochondrial genomes in the unique regions of CMS lines are often considered to be the genes controlling the CMS trait. In this study, twenty-seven 138A specific ORFs that encoded ≥100 amino acids were identified (Supplementary Materials Table S2), of which 11 ORFs were located in the unique regions of 138A with full length or partial sequences (Supplementary Materials Table S3).

#### 2.4.2. Analyses of Chimeric Structures, Co-Transcript Event, and Transmembrane Domain

**Figure 4:**
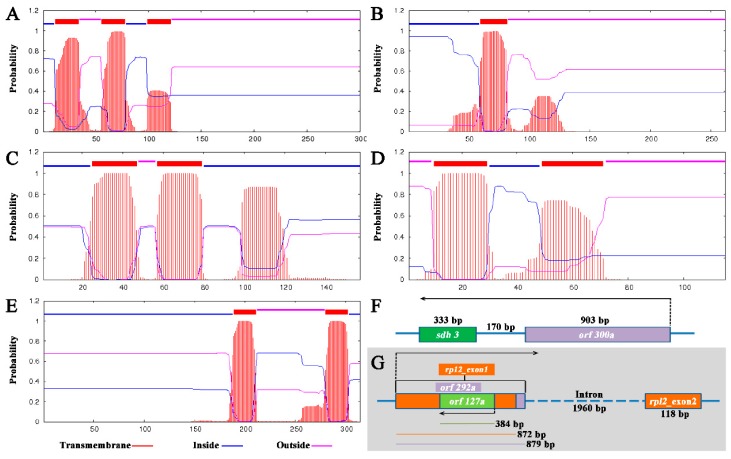
Chimeric structures, co-transcribe event, and transmembrane domain of the specific ORFs in 138A. (**A**–**E**): Locations and probabilities of the transmembrane domains of the gene products of *orf300a* (**A**); *orf262a* (**B**); *orf157a* (**C**); *orf115b* (**D**); and *orf314a* (**E**). (**F**) Co-transcribe structure of *orf300a* and *sdh3*; (**G**) chimeric structure of *orf292a* and *rpl2*_-_exon1, a long horizontal arrow shows orientation of transcription.

**Figure 5:**
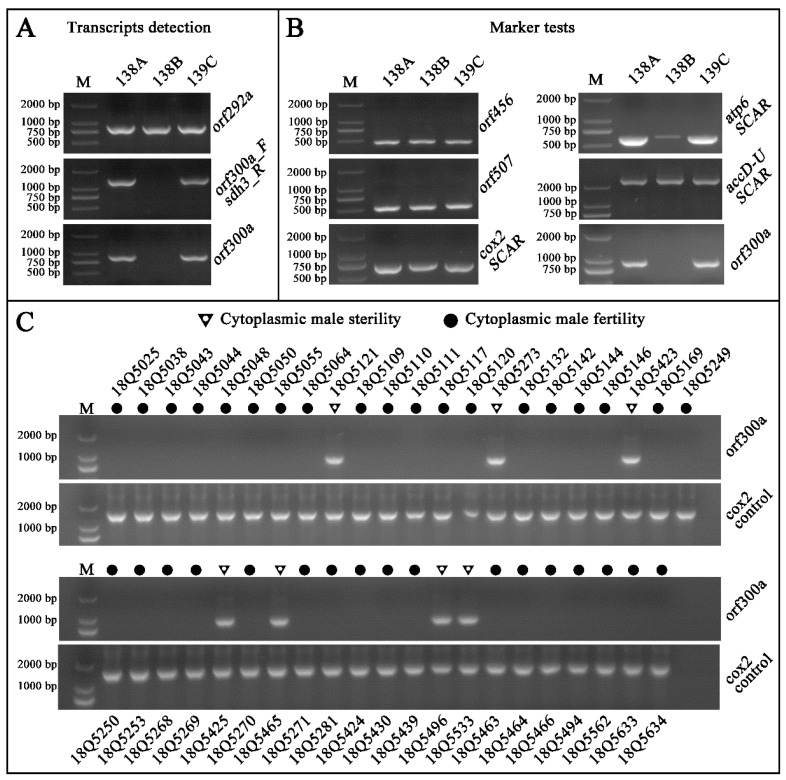
Results of PCR amplification. (**A**) Transcript detection of *orf292a*, co-transcript detection of *orf300a-sdh3*, transcript detection of *orf300a*; (**B**) CMS makers (orf456, orf507, cox2 SCAR, atp6 SCAR, accD-U SCAR, orf300a) test in 138A, 138B, 139C. (**C**) CMS maker orf300a tests in 43 pepper inbred lines, cox2 positive control.

**Figure 6:**
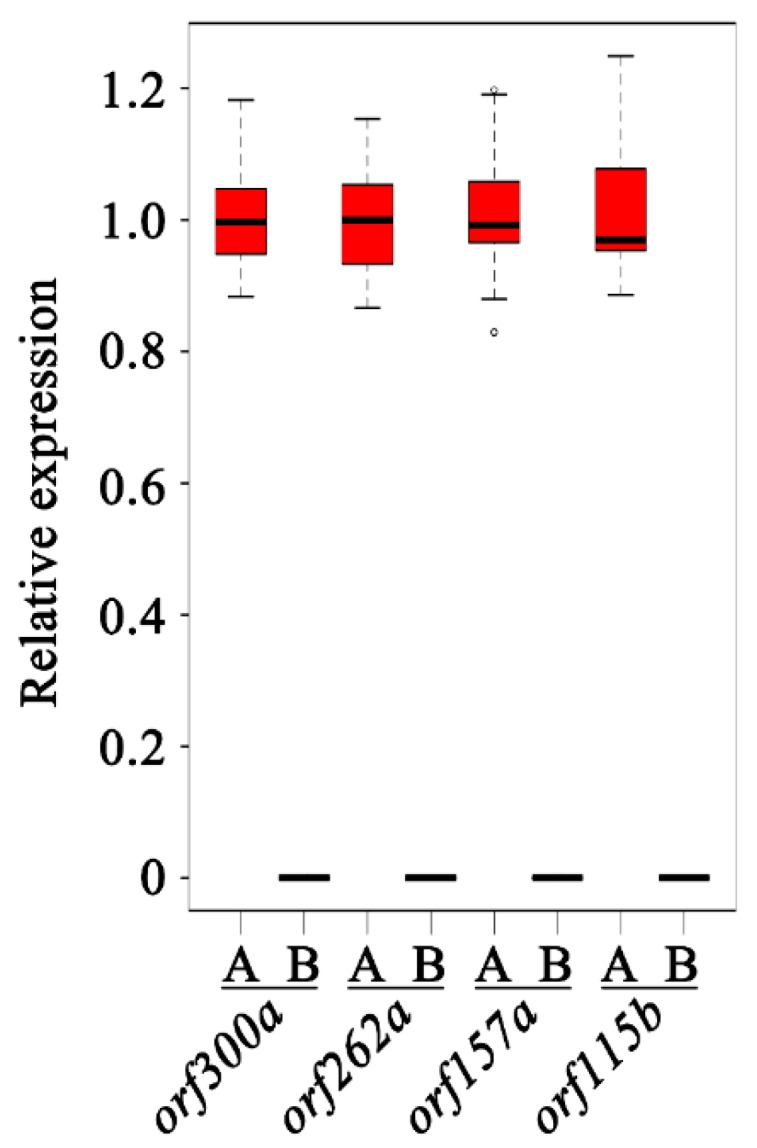
Transcriptional level of four 138A specific ORFs (*orf262a*, *orf300a*, *orf157a*, *orf115b*) in CMS line 138A and maintainer line 138B. Expression value the four ORFs in 138A was set as 1. *UBI-3* was employed as the internal control. (**A**) and (**B**) represented lines 138A and 138B, respectively.

It is known that most CMS and CMS candidate genes feature chimeric structures and/or co-transcribing with functional genes. Thus, chimeric structures and co-transcripts were analyzed with the 138A specific ORFs. To the chimeric structure, only one chimeric structure was predicted. This chimeric structure was found in *orf292a*, which contained whole *rpl2* exon1 and a 7 bp sequence of unknown origin at the 3′ end (Figure 4G). *Rpl2* encodes a ribosomal protein large subunit. The existence of the *orf292a* transcript was confirmed by RT-PCR and sequencing (Figure 5A). Another co-transcript event was only confirmed in *orf300a* and the nearby gene *sdh3*. This transcript consisted of the full length of *orf300a* and *sdh3* as well as the 171 bp intergenic sequence (Figure 4F). The co-transcript was also confirmed by RT-PCR (primers: *orf300a*_F/*sdh3*_R) and sequencing (Figure 5A). As transmembrane domain is another characteristic of the CMS genes, we performed structural analysis of 138A specific novel ORFs. The following five ORFs (*orf157a*, *orf314a*, *orf262a*, *orf300a*, and *orf115b*) were examined for the presence of transmembrane domains (Supplementary Materials Table S2, Figure 4A–E). Also, the expression patterns of four candidate genes, including *orf157a*, *orf262a*, *orf300a*, and *orf115b*, were also analyzed in anthers of 138A and 138B using real-time PCR (Figure 6). Results showed that almost none of those four genes were expressed in 138B, suggesting those genes expressed at very low level in the maintainer line (Figure 6).

### 2.5. CMS Marker Development and Testing

Identification of the CMS seedlings in the offspring is very important for the development of CMS lines. Molecular markers for the CMS trait are useful tools for accelerating the above process as well as the application of CMS in the F_1_ seed production. So far, at least five molecular markers have been developed for the identification of pepper CMS, including atp6 SCAR [30], cox2 SCAR [30], orf456 [32], orf507 [33], and acc D–U [34]. However, in our CMS lines and populations, they did not co-segregate with the CMS trait. Particularly, marker atp6 SCAR could not only be amplified in 138A, and 139C (contains sterile cytoplasm and the restorer of fertility (*Rf*) gene) lines, but also could be amplified in 138B (Figure 5B). Markers cox2 SCAR, orf456, orf507, and acc D–U did not show polymorphism among 138A, 138B, and 139C lines (Figure 5B). BLASTs result also supported the above observation that the primers sequences of marker cox2 SCAR, orf456 and orf507 could match the mitochondrial genomes of 138A and 138B very well. For marker acc D-U, primers did not align to genome sequences of 138A or 138B. Moreover, when those markers were used in the 43 pepper inbred lines which were developed in our lab from our CMS source (Supplementary Materials Table S9), no polymorphism was observed. Therefore, we tried to develop new markers for identifying the pepper CMS trait. Based on the unique ORFs in 138A, marker orf300a was developed. Further confirmation of this new marker was performed in the 43 pepper inbred lines which were developed in our lab and the male-sterile phenotypes of those lines were already known (Supplementary Materials Table S9). The results shown in Figure 5C indicated that the marker did completely co-segregate with the CMS trait, indicating this marker can be used to screen CMS plants which were originated from our CMS sources. 

## 3. Discussion

### 3.1. 138A and FS4401 May Originate from a Common Female Ancestor, but Their CMS May Originate Separately

Through sequencing and de novo assembling, completed mitochondrial genomes of a pepper CMS line of Chinese origin (138A) and its maintainer line (138B) were constructed in this study. The mitochondrial genome sequence and structure of 138A was quite similar to that of FS4401, a previously reported Korean pepper CMS line, with the sequence variation rate and structure variation rate less than 2% and 1%, respectively. Moreover, a similar phenomenon was also observed between the maintainer line 138B and a Korean local variety “Jeju”, with the sequence variation rate and structure variation rate less than 2% and 1%, respectively. For the CMS lines, although 138A was developed from a CMS plant which was discovered in the experimental field in China in 1986 [35] and the FS4401 was developed in Korea, their mitochondrial genome sequence and structure were highly similar to each other, suggesting they may originate from a common female ancestor. However, SNPs and InDels were identified between 138A and FS4401, and moreover, no difference was observed in *orf507* between 138A and 138B, which is considered to be the gene controlling the CMS in FS4401, indicating that CMS of 138A was not transformed from FS4401 or its recent female parent, and vice versa. In regard to the maintainer line 138B and the Korean local variety “Jeju”, it is surprising that their mitochondrial genomes were also highly similar to each other. 138B is an inbred line of *C. annuum* var. “Shanghaiyuan”, which is an old local sweet pepper variety in the southeast of China. In regard to *C. annuum* var. “Jeju”, according to the previous publications, it is a Korean local hot pepper variety [36]. Therefore, it is plausible that 138B and “Jeju” are two different varieties and it can be presumed that the variation of mitochondria genome in *C. annuum* or at least in some *C. annuum* varieties is small. A similar phenomenon was also reported in other *Solanaceae* species. In tomato the mitochondrial genome sequence of a wild tomato, *Solanum pennellii*, is 98% similar to that of the *S. lycopersicum* [37]. In conclusion, the mitochondrial genomes of 138A and FS4401 highly likely have been inherited from a common female ancestor but their CMS originated separately.

### 3.2. Candidate Gene Selection for the CMS Trait in 138A

Cytoplasmic male sterility is an important trait for the hybrid seed production of both cereal and horticultural crops. So far, it has been confirmed that the CMS is not only caused by the SNPs and InDels, but also attributed to the rearrangement and recombination of the mitochondrial genome, which usually leads to the generation of new ORFs, chimeric structures, and co-transcribe events. Thus, for CMS candidate gene selection, the mitochondrial genome sequence variations as well as the mutations caused by the genome structural variations should all be considered. SNPs and indels leading to non-synonymous mutations and/or missing aas were detected in eight ORFs (Table 1), two of which were in the following known genes, *orf25* and *orfB*. Similarly, in tobacco a truncated *orf25* caused by an SNP was considered a strong candidate gene for CMS [38,39,40]. Thus, those eight genes with SNPs and InDels should be selected as potential candidate genes underlying the CMS. Also, new ORFs, chimeric structures, co-transcribe, and transmembrane domains are also important reasons for the CMS. For example, *orf463*, *orf125*, and *orf138*, which are the new ORFs generated by the rearrangement and recombination of the mitochondrial genome, are reasons for the CMS-DCGMS in radish, CMS-Kos in radish, and CMS-Ogu in Brassica, respectively [19,41,42]; in sorghum, *orf107* encoding a chimeric protein with a segment of *ATP9* at the N terminus is confirmed to cause CMS-A3 [43]; in sunflower, *orf522* which is co-transcribed with the *ATPA* gene has been certified to induce the CMS [44]. In this work, 27 138A unique ORFs were identified which were all possible to cause the CMS and should be considered as the potential candidate genes. Furthermore, based on previous studies, most of the CMS genes have the chimeric structure and/or transmembrane domains, which lead to the disturbance of the normal function of mitochondria and finally cause the CMS. Meanwhile, those CMS genes are quite often found to be located on the unique regions of the CMS mitochondrial genomes. Therefore, those conditions were all considered in the further screen of the candidate genes in this study. Finally, 14 138A specific ORFs were found to have several or at least one of the following features: location on the unique region, chimeric structure, co-transcription, and transmembrane domain (Table 1). Among those 14 ORFs, *orf292a* was predicted to only have a chimeric structure; *orf115b* and *orf157a* were only considered to harbor transmembrane domains in their encoding products; *orf262a* and *orf314a* were predicted to not only have transmembrane domains, but also be located on the 138A unique regions; *orf300a*, which is located on the unique region of 138A, was predicted to not only have the transmembrane domains, but also co-transcribe with the *sdh3* gene. Thus, all those 14 ORFs should be considered as the candidate genes for CMS in 138A. As *orf300a* met most of the conditions mentioned above, it was further selected as a strong candidate gene. Besides *orf300a*, *orf314a* was also considered to be a strong candidate for CMS gene. The sequence of *orf314a* in 138A was highly similar (99%) to that of the *atp6* in 138B; however, the length of *orf314a* (945 bp) was 351 bp shorter than that of the *atp6*. Meanwhile, *atp6a* in 138A was also highly similar (99%) to that of *atp6* in 138B, but the length of *atp6a* (855 bp) was 441 bp shorter than of *atp6* (Supplementary Materials Figure S1). *atp6* encodes the *ATP6* subunit of the mitochondrial ATP synthase and has been confirmed to be involved in the control of CMS. Therefore, it is high likely that *orf314a* and *atp6a* in 138A originated from the *atp6* gene. During the rearrangement and recombination of the mitochondrial genome, the original *atp6* in 138A did break into two pieces, and later, one piece evolved into *apt6a* and the other evolved into *orf314a*. This variation may lead to the disturbance of the normal function of *atp6* and cause the CMS in 138A. Similar phenomenon was also reported by Kim et al. [31]. Finally, in this study, two ORFs, *orf300a* and *orf314a*, were selected as strong candidates for the CMS controlling gene in 138A. However, in the previous publication, *orf507* was considered as a strong candidate gene for CMS in FS4401, because it was only found in the CMS line but not the MF line. Moreover, the short form of *orf507*, named *orf456*, has been confirmed to cause CMS via ectopic expression. In this work, *orf507* was annotated in the mitochondrial genomes of 138A and 138B, and no sequence variation was found between the CMS and maintainer lines (Supplementary Materials Figure S2), suggesting *orf507* should not be the candidate gene for CMS.

## 4. Materials and Methods

### 4.1. Plant Materials

A pepper CMS line 138A and its maintainer line 138B were used in this study. 138A and 138B are in the sweet pepper “Shanghaiyuan” background. The CMS trait of 138A was transferred from an inbred line which originated from a CMS plant that was discovered in the production field of variety “8633” in 1986. Approximately 1000 seeds were sown in a growth chamber with 28 °C, 70% humidity, 16 h light/8 darkness. Tender roots of the two lines were collected at the four-leaf stage, washed with tap water and frozen with liquid nitrogen, followed by storage at −80 °C. 

### 4.2. Mitochondrial DNA Extraction

Mitochondrial DNA was extracted using the GENMED large number of plant tissue mitochondrial DNA extraction kit (Genmed Scientifics Inc., Arlington, MA, USA). Pepper tender roots (60 g) were ground in a mortar under liquid nitrogen. The powdered tissues were then homogenized with 500 mL homogenization buffer (supply by the kit) in a glass homogenizer. The quality of DNA was assessed using NanoDrop2000, qubit3.0, and 0.8% agarose gel electrophoresis.

### 4.3. Sequencing and Assembling of the Mitochondrial Genome

One microgram of the purified DNA was fragmented to construct 430-bp short-insert libraries according to the manufacturer’s instructions (Illumina, Hercules, CA, USA), and then sequenced on the Illumina Hiseq 4000 platform [45]. The high-molecular weight DNA was purified and used for PacBio library prep, BluePippin size selection, and then sequenced on the Sequel Sequencer.

Prior to assembly, the Illumina raw reads were filtered to remove the reads with adaptors, the reads showing a quality score below 20(Q < 20), the reads containing a percentage of uncalled based (“N” characters) equal or greater than 10%, and the duplicated sequences. The mitochondrial genome was reconstructed using a combination of the Pacbio Sequel data and the Illumina Hiseq data, and the following three steps were used to assemble the mitochondrial genomes. First, the genome framework was assembled by combing the Illumina and Pacbio data using SPAdes v3.10.1 [46]. Second, the assembly was verified and the circular or linear characteristics of the mitochondrial genome were completed, filling the gaps if any. Third, the clean reads were mapped to the assembled mitochondrial genome to correct the wrong bases and the presence of any insertion and deletion was judged. Finally, the complete mitochondrial genome sequences of 138A (CMS cytoplasm) and 138B (normal cytoplasm) were acquired and deposited in the GenBank nucleotide sequence database.

### 4.4. Genome Component Analysis

#### 4.4.1. Gene Annotation and Identification of ORFs

The mitochondrial genes were annotated using homology alignments and de novo prediction. EVidenceModeler v1.1.1 was used to integrate the gene set [47]. The transfer RNA (tRNA) and ribosomal RNA (rRNA) genes were predicted by tRNAscan-SE [48] and rRNAmmer 1.2 [49], respectively. A whole mitochondria genome BLAST [50] (basic local alignment search tool) search (E-value ≤ 1 × 10^−5^, minimal alignment length percentage ≥ 40%) was performed against the following five databases: KEGG (Kyoto Encyclopedia of Genes and Genomes) [51,52,53], COG (Clusters of Orthologous Groups) [54,55], NR (Non-Redundant Protein Database databases), Swiss-Prot [56], and GO (Gene Ontology) [57]. The circular map of the mitochondrial genome was drawn using OrganellarGenomeDRAW v1.2 [58]. The prediction of the ORFs was undertaken using the software ORF Finder (http://www.ncbi.nlm.nih.gov/gorf/gorf.html) if the hypothetical proteins encoded by the ORFs were longer than 100 amino acids. The names of the ORFs were given based on the number of amino acids that they encoded. Orfxx + suffix a/b/c: xx represents the length of the amino acid sequence of the orf, while the suffix a/b/c represents the same length, but different genes; orfxx + suffix -1/-2/-3: xx represents the length of the amino acid sequence of ORF, and the suffix -1/-2/-3 represents the duplicated genes of the same length.

The presence of a transmembrane domain in each hypothetical protein was predicted using TMHMM server v.2.0 (http://www.cbs.dtu.dk/services/TMHMM/).

#### 4.4.2. Sequence Comparison between 138A and 138B

In order to identify the syntenic sequence blocks and their translocation and inversion, a whole-genome synteny analysis was performed between 138A and 138B using the software programs MUMmer v3.23 and LASTZ v1.03.54. A single nucleotide polymorphism (SNP) was identified between 138A and 138B using the software programs MUMmer and BLAT v35. The size of the insertion–deletion (InDel) sequence ranging from 1 to 10 bp was identified using the software programs LASTZ v1.03.54, BWA, and SAMtools. The structural variation (SV) of the two genomes was identified using the software programs MUMmer v3.23 and LASTZ v1.03.54.

#### 4.4.3. RT–PCR, Real-time PCR, and Sequencing of PCR Products

Total RNA was isolated from different tissues and organs (root; stems; leaf; petals, anther, sepals and ovary at different times) of 138A, 138B, and 139C using an RNA extraction kit (SV total RNA isolation system, Promega), and the resulting RNA was reverse transcribed using the PrimeScript^TM^ RT Reagent Kit (TaKaRa Bio Inc., Kusatsu, Shiga, Japan) to obtain 20 μL of cDNA solution. The RT-PCR was performed in a 50-μL reaction volume containing 2 μL template cDNA, 25 μL high fidelity DNA polymerase (TaKaRa Bio Inc., Kusatsu, Shiga, Japan), 2 μL forward primer (10 μM), 2 μL reverse primer (10 μM), and 19 μL deionized water. The primers used in this study are listed in Supplementary Table S10. The RT-PCR was conducted using the following thermal cycles: initial denaturation at 94 °C for 5 min; 28 cycles of 98 °C for 10 s, 55 °C for 15 s, and 72 °C for 1 min; then followed a final 5 min extension at 72 °C. To add poly-A tails to PCR products, 10 μL Taqmix was added to 50 μL PCR product and the mixture was incubated at 72 °C for 30 min. The amplification products were detected by electrophoresis on a 1.5% agarose gel (containing gold view nucleic acid dye) and analyzed by UVI gel imaging system. The PCR products were purified using an Agarose Gel DNA Recovery Kit (GENERAY BIOTECH, Shanghai, China). The purified PCR product (4 μL) was mixed with 1 μL PMD19-T (TaKaRa Bio Inc., Kusatsu, Shiga, Japan) and 5 μL ligase mix and incubated for 10 h at 16 °C. Subsequently, the ligated product was sequenced by ABI PRISM 3730XL Analyzer (Applied Biosystems, Foster City, CA, USA).

The real-time PCR was performed using the TB Green^TM^ Premix Ex Taq^TM^ (TaKaRa Bio Inc., Kusatsu, Shiga, Japan), following manufacturer’s instructions on an ABI 7500 real-time PCR system. The thermocycling conditions were set as follows: 95 °C for 30 s, 40 cycles of 95 °C for 5 s, and 60 °C for 34 s. Expression results were plotted based on 2^−ΔΔ*C*t^ method. Boxplot was generated via boxplot() in R.

## 5. Conclusions

Cytoplasmic male sterility is a very important phenotypic trait in pepper breeding. The present study reveals the complete genome sequences of the mitochondria in a pepper CMS line 138A cytoplasm and its maintainer line 138B normal cytoplasm. Those sequences can be found and downloaded in the GenBank nucleotide sequence database (https://www.ncbi.nlm.nih.gov/genbank/) under the accession numbers of MK225636 and MK225637, respectively. By comparing the two mitochondrial genomes, several ORFs (*orf300a*, *orf314a*, *orf262a*, *orf292a*, *orf157a*, *orf115b*) that might be related to CMS, among them, *orf300a* and *orf314a*, were selected as strong candidates for the CMS controlling gene in 138A. In addition, orf300a has been successfully applied to CMS material screening as a molecular marker.

## Figures and Tables

**Table 1:** Features of the candidate genes controlling the CMS.

Features of the ORF	ORF ID
SNP/InDel	*orf229a*, *orf138a*, *orf337a*, *orf675a*, *orf249a*, *orf104c*, *orfB*, *orf25*
On the unique region	*orf108g*, *orf132a*, *orf314a*, *orf262a*, *orf165a*, *orf338a*, *orf244a*, *orf100b*, *orf300a*, *orf119a*, *orf100a*
Chimeric structure/ co-transcription	*orf292a*, *orf300a*
Transmembrane domain	*orf300a*, *orf314a*, *orf157a*, *orf115b*, *orf262a*

## Supplementary Materials

Supplementary materials can be found at http://www.mdpi.com/1422-0067/20/3/578/s1.
