# Supplementary material for: Candidate Gene Selection for Cytoplasmic Male Sterility in Pepper (Capsicum annuum L.) through Whole Mitochondrial Genome Sequencing
Source: Int J Mol Sci. 2019 Jan 29;20(3):578. doi: 10.3390/ijms20030578 (PMC6386957; doi:10.3390/ijms20030578)
Supplement: Supplementary file 1 [file ijms-20-00578-s001.pdf]

Table S1 The results of genetic prediction of 138A and 138B

Gene ID in 138A	mRNA start (bp)	mRNA end (bp)	Gene ID in 138B	mRNA start (bp)	mRNA end (bp)
atp1	366454	367983	*atp1*	139478	141007
atp6a	18311	19165	*atp6*	216911	218206
atp9	361077	361310	*atp9*	134083	134316
ccmB	419104	419724	*ccmB*	337541	338161
ccmC	127246	127998	*ccmC*	65687	66439
ccmFc	449048	451309	*ccmFc*	320141	322402
ccmFN	216352	218121	*ccmFN*	167657	169426
cob	208057	209238	*cob*	159362	160543
cox1	120831	122414	*cox1*	71271	72854
cox2	248099	249780	*cox2*	498662	500343
cox3	309798	310595	*cox3*	269485	270282
mat-R	282583	284559	*mat-R*	99831	101807
nad1	9114	427026	*nad1*	25613	298076
nad2	299505	386510	*nad2*	116758	375645
nad3	332402	332758	*nad3*	292136	292492
nad4	103291	111977	*nad4*	81702	90388
nad4L	163495	163797	*nad4L*	29827	30129
nad5	154710	395190	*nad5*	38923	364386
nad6	93296	93952	*nad6*	412957	413613
nad7	481079	486370	*nad7*	1875	7142
nad9	409327	409899	*nad9*	347376	347948
orf100a	305194	305496	*orf100c-1*	98113	98415
orf100b	274128	274430	*orf100c-2*	411343	411645
orf100c	95264	95566	*orf100d*	426225	426527
orf100d	80401	80703	*orf101a*	362046	362351
orf101a	394936	395241	*orf101b*	232387	232692
orf101b	3825	4130	*orf102a*	501452	501760
orf102a	250889	251197	*orf102b*	361110	361418
orf102b	395869	396177	*orf102c*	328647	328955
orf102c	457545	457853	*orf102d*	427455	427763
orf102d	79165	79473	*orf102e*	230208	230516
orf102e	6001	6309	*orf102f*	496078	496386
orf102f	245515	245823	*orf102g*	173340	173648
orf102g	222035	222343	*orf102h*	53183	53491
orf102h	140163	140471	*orf102i*	59215	59523
orf102i	134152	134460	*orf102j*	68932	69240
orf102j	124445	124753	*orf102k*	428596	428904
orf102k	78024	78332	*orf102l*	470324	470632
orf102l	36428	36736	*orf102m*	366458	366766
orf103a	312999	313310	*orf103a*	272695	273006
orf103b	394684	394995	*orf103b*	362292	362603
orf103c	417061	417372	*orf103c*	339893	340204

orf103d	397354	397665	*orf103d*	359622	359933
orf103e	187223	187534	*orf103e*	402916	403227
orf104a	325861	326175	*orf103f-1*	210133	210444
orf104b	453552	453866	*orf103f-2*	260647	260958
orf104c	332923	333237	*orf104a*	285595	285909
orf104d	110225	110539	*orf104b*	324645	324959
orf105a	218067	218384	*orf104d*	83140	83454
orf105b	290111	290428	*orf104e*	191898	192212
orf106a	321777	322097	*orf105a*	169372	169689
orf106b	330547	330867	*orf105b*	107364	107681
orf106c	409945	410265	*orf106a*	281446	281766
orf106d-1	174058	174378	*orf106b*	290281	290601
orf106d-2	468275	468595	*orf106c*	347010	347330
orf107a	28558	28881	*orf106d-1*	198553	198873
orf107b	290648	290971	*orf106d-2*	249067	249387
orf107c	73915	74238	*orf107a*	478178	478501
orf107d	124563	124886	*orf107b*	107901	108224
orf108a	57463	57789	*orf107d*	68799	69122
orf108b-1	183430	183756	*orf108a*	449168	449494
orf108b-2	477647	477973	*orf108b*	511271	511597
orf108c	395417	395743	*orf108c*	361544	361870
orf108d	372473	372799	*orf108d-1*	145506	145832
orf108e	90056	90382	*orf108d-2*	391088	391414
orf108f	143079	143405	*orf108e*	416527	416853
orf108g	23578	23904	*orf108f*	50249	50575
orf108h	12738	13064	*orf108h*	223453	223779
orf109a	309132	309461	*orf109a*	268819	269148
orf109b	31888	32217	*orf109b*	474843	475172
orf109c	491447	491776	*orf109c*	12219	12548
orf110a	33111	33443	*orf110a*	473617	473949
orf110b	212143	212475	*orf110b*	163448	163780
orf110c	202103	202435	*orf110c*	153396	153728
orf111a	311885	312220	*orf110d*	483155	483487
orf111b	14408	14743	*orf111a*	271581	271916
orf112a	100356	100694	*orf111b*	221774	222109
orf113a	62908	63249	*orf112a-1*	92985	93323
orf114a	62487	62831	*orf112a-2*	406215	406553
orf114b	336995	337339	*orf113a*	443708	444049
orf115a	448241	448588	*orf114a*	444126	444470
orf115b	421643	421990	*orf114b-1*	201328	201672
orf115c	110223	110570	*orf114b-2*	251842	252186
orf117a-1	116220	116573	*orf115a*	319334	319681
orf117a-2	272073	272426	*orf115c*	83109	83456
orf117b	431927	432280	*orf115d*	241791	242138

orf117c	93633	93986	*orf116a*	73745	74095
orf118a	308547	308903	*orf117a*	77106	77459
orf119a	302441	302800	*orf117b*	302987	303340
orf119b	310189	310548	*orf117c*	412923	413276
orf119c	306816	307175	*orf118a*	268234	268590
orf121a	346558	346923	*orf119b*	269876	270235
orf122a	285780	286148	*orf119c*	266503	266862
orf123a	64629	65000	*orf121a*	119554	119919
orf123b	69409	69780	*orf121b*	53503	53868
orf124a	78654	79028	*orf122a*	103028	103396
orf124b	114382	114756	*orf123a*	441947	442318
orf125a	368798	369175	*orf123b*	437156	437527
orf125b-1	176259	176636	*orf124a*	427900	428274
orf125b-2	470476	470853	*orf124b*	78923	79297
orf125c	482354	482731	*orf125a-1*	141822	142199
orf125d	425958	426335	*orf125a-2*	387404	387781
orf125e	128376	128753	*orf125b*	504090	504467
orf125f	116008	116385	*orf125c*	3150	3527
orf126a	501985	502365	*orf125d*	297008	297385
orf126b	371594	371974	*orf125e*	64932	65309
orf127a	186153	186536	*orf125f*	77294	77671
orf128a	203320	203706	*orf128a*	154613	154999
orf128b	420221	420607	*orf128b*	336658	337044
orf128c	485984	486370	*orf128c*	6756	7142
orf129a	9109	9498	*orf129a*	227019	227408
orf131a	297998	298393	*orf129b-1*	144618	145007
orf131b	420262	420657	*orf129b-2*	390200	390589
orf131c	104430	104825	*orf130a*	403914	404306
orf134b	254710	255108	*orf131a*	115251	115646
orf132b	502655	503053	*orf131b*	336608	337003
orf132c	63926	64324	*orf131c*	88854	89249
orf133a	352223	352624	*orf132b*	237692	238090
orf133b	395760	396161	*orf132c*	442623	443021
orf133c	10212	10613	*orf133a*	125219	125620
orf134a-1	173716	174120	*orf133b*	361126	361527
orf134a-2	467933	468337	*orf133c*	225904	226305
orf134b	300670	301074	*orf134a-1*	198211	198615
orf134c	108846	109250	*orf134a-2*	248725	249129
orf135a	248099	248506	*orf134b*	117923	118327
orf137a	166115	166528	*orf134c*	84429	84833
orf138a	95973	96389	*orf135a*	498662	499069
orf140a	421216	421638	*orf137a*	27096	27509
orf141a	280940	281365	*orf138a-1*	97290	97706
orf142a	416055	416483	*orf138a-2*	410520	410936

orf144a	89366	89800	*orf142a*	340790	341218
orf145a	363072	363509	*orf144a*	417109	417543
orf146a	389323	389763	*orf145a*	136078	136515
orf147a	48262	48705	*orf146a*	367544	367984
orf147b	154914	155357	*orf147a*	458346	458789
orf149a-1	471244	471693	*orf147b*	38297	38740
orf149a-2	177027	177476	*orf150a*	292136	292588
orf150a	332402	332854	*orf152b*	444737	445195
orf152a	324225	324683	*orf152c-1*	96105	96563
orf152b	61762	62220	*orf152c-2*	409335	409793
orf152c	97116	97574	*orf152d*	40664	41122
orf152d	152532	152990	*orf154a*	172062	172526
orf154a	220757	221221	*orf158a*	108348	108824
orf157a	119467	119940	*orf161a*	38370	38855
orf158a	291095	291571	*orf162a*	508356	508844
orf161a	154799	155284	*orf163a*	426873	427364
orf162a-1	180515	181003	*orf164a*	307622	308116
orf162a-2	474732	475220	*orf166a*	2217	2717
orf163a	79564	80055	*orf166b*	385421	385921
orf164a	436562	437056	*orf168a*	500566	501072
orf165a	271323	271820	*orf169a*	314726	315235
orf166a	481421	481921	*orf171a*	497898	498413
orf168a	250003	250509	*orf171b*	87913	88428
orf169a	443642	444151	*orf172a*	66844	67362
orf171a	247335	247850	*orf178a*	427851	428387
orf171b	105251	105766	*orf185a*	89861	90418
orf178a	78541	79077	*orf185b*	220934	221491
orf184a	126323	126877	*orf187a*	220004	220567
orf185a	103261	103818	*orf188a*	436062	436628
orf185b	15026	15583	*orf188b*	162199	162765
orf187a	15950	16513	*orf190a*	80740	81312
orf188a	70308	70874	*orf204a*	111039	111653
orf190a	112367	112939	*orf204b*	488433	489047
orf204a	293786	294400	*orf208a*	321776	322402
orf204b	237881	238495	*orf214a*	373419	374063
orf208a	450683	451309	*orf224a*	151963	152637
orf214a	383255	383899	*orf226a*	504637	505317
orf224a	200670	201344	*orf229a*	475403	476092
orf229a	30968	31657	*orf237a*	4448	5161
orf237a	483664	484377	*orf240a*	77818	78540
orf240a	115139	115861	*orf242a*	279403	280131
orf242a	319734	320462	*orf249a*	23773	24522
orf244a	273300	274034	*orf261a*	335264	336049
orf249a	169102	169851	*orf264a-1*	141062	141856

orf262a	271069	271857	*orf264a-2*	386644	387438
orf264a	368038	368832	*orf267a*	21030	21833
orf267a	500246	501049	*orf279a*	411761	412600
orf279a	94309	95148	*orf293a-1*	90486	91367
orf292a	185862	186740	*orf293a-2*	875	1756
orf293a-1	102312	103193	*orf294a*	320141	321025
orf293a-2	480079	480960	*orf295a*	403710	404597
orf294a	449048	449932	*orf301a*	127413	128318
orf300a	302143	303045	*orf337a*	44076	45089
orf301a	354417	355322	*orf353a*	239901	240962
orf314a	262814	263758	*orf399a*	363193	364392
orf337a	148565	149578	*orf427a*	277140	278423
orf338a	272261	273277	*orf470a*	385177	386589
orf399a	392895	394094	*orf473a*	238532	239953
orf430a	317434	318726	*orf481a-1*	208221	209666
orf484a	51001	52455	*orf481a-2*	258735	260180
orf498a	48702	50198	*orf484a*	454596	456050
orf515a	53230	54777	*orf498a*	456853	458349
orf543a	144640	146271	*orf543a*	47383	49014
orf545a	295666	297303	*orf545a*	112919	114556
orf559a	245765	247444	*orf551a*	452180	453835
orf603a	216352	218163	*orf559a*	496328	498007
orf675a	149724	151751	*orf603a*	167657	169468
orf750a	198392	200644	*orf675a*	41903	43930
orfB	311237	311698	*orf750a*	149685	151937
orfX	420262	421113	*orfB*	270924	271394
rpl10	189100	189579	*orfX*	336152	337003
rpl16	247374	247850	*rpl10*	400909	401388
rpl2	185862	188811	*rpl16*	497937	498413
rpl5	210894	211448	*rpl2*	401677	404597
rps10	11900	13007	*rpl5*	162199	162753
rps12	331976	332353	*rps10*	223510	224617
rps13	168542	168892	*rps12*	291710	292087
rps19	244385	244669	*rps13*	24732	25082
rps3	244683	247444	*rps19*	494948	495232
rps4	153253	154290	*rps3*	495246	498007
sdh3	301640	301972	*rps4*	39364	40401
sdh4	309493	309870	*sdh3*	118893	119225
orf25	162719	163306	*sdh4*	269180	269557
			orf25	30318	30914

Table S2 The specific ORFs in 138A and their transmembrane domain number

Gene ID	mRNA start (bp)	mRNA end (bp)	No. of transmembrane domain
orf108g	23578	23904	0
orf515a	53230	54777	0
orf107c	73915	74238	0
orf157a	119467	119940	2
orf184a	126323	126877	0
orf149a-2	177027	177476	0
orf292a	185862	186740	0
orf127a	186153	186536	0
orf132a	254710	255108	0
orf314a	262814	263758	2
orf262a	271069	271857	1
orf165a	271323	271820	0
orf338a	272261	273277	0
orf244a	273300	274034	0
orf100b	274128	274430	0
orf141a	280940	281365	0
orf300a	302143	303045	3
orf119a	302441	302800	0
orf100a	305194	305496	0
orf430a	317434	318726	0
orf152a	324225	324683	0
orf104c	332923	333237	0
orf126b	371594	371974	0
orf140a	421216	421638	0
orf115b	421643	421990	2
orf149a-1	471244	471693	0
orf126a	501985	502365	0

Table S3 The location information of 138A unique regions and the ORFs in it

No.	start (bp)	end (bp)	size (bp)	orf name	orf start (bp)	orf end (bp)
1	19114	23689	4576	*orf108g*	23578	23904
2	174859	175164	306	*NA*		
3	251956	257728	5773	*orf132a*	254710	255108
4	263745	269224	5480	*orf314a*	262814	263758
5	270983	280625	9643	*orf262a*	271069	271857
				orf165a	271323	271820
				orf338a	272261	273277
				orf244a	273300	274034
				orf100b	274128	274430
6	302060	305384	3325	*orf300a*	302143	303045
				orf119a	302441	302800
				orf100a	305194	305496
7	333326	334920	1595	*NA*		
8	346229	346265	37	*NA*		
9	464064	464268	205	*NA*		
10	469076	469381	306	*NA*		
11	501404	501422	19	*NA*		

Table S4 The specific ORFs in 138B and their transmembrane domain number

Gene ID	mRNA start (bp)	mRNA end (bp)	No. of transmembrane domain
orf121b	53503	53868	0
orf172a	66844	67362	0
orf116a	73745	74095	2
orf129b-1	144618	145007	0
orf188b	162199	162765	0
orf104e	191898	192212	0
orf481a-1	208221	209666	0
orf103f-1	210133	210444	0
orf431a	216911	218206	5
orf473a	238532	239953	6
orf353a	239901	240962	6
orf115d	241791	242138	0
orf481a-2	258735	260180	0
orf103f-2	260647	260958	0
orf427a	277140	278423	0
orf261a	335264	336049	2
orf102m	366458	366766	0
orf470a	385177	386589	1
orf166b	385421	385921	0
orf129b-2	390200	390589	0
orf295a	403710	404597	0
orf130a	403914	404306	0
orf551a	452180	453835	0
orf110d	483155	483487	0
orf226a	504637	505317	0
orf108b	511271	511597	0

Table S5 The location information of 138B unique regions and the ORFs in it

No.	start (bp)	end (bp)	size (bp)	orf name	orf start (bp)	orf end (bp)
1	23622	23738	117	*NA*		
2	98479	98523	45	*NA*		
3	184124	194592	10469	*orf104e*	191898	192212
4	207737	211824	4088	*orf481a-1*	208221	209666
				orf103f-1	210133	210444
5	238448	241795	3348	*orf473a*	238532	239953
				orf353a	239901	240962
				orf115d	241791	242138
6	242701	245060	2360	*NA*		
7	258251	262181	3931	*orf481a-2*	258735	260180
				orf103f-2	260647	260958
8	293060	293089	30	*NA*		
9	382300	384754	2455	*orf470a*	385177	386589
10	384968	386081	1114	*orf166b*	385421	385921
11	453092	453146	55	*orf551a*	452180	453835
12	502519	502995	477	*NA*		

Table S6 The results of SNP annotation with 138A as the reference sequence

Sample ID	CDS					Intergenic	Total
	Start- nonsyn	Stop- nonsyn	Premature- stop	Synonymous	Nonsynonymous		
138B	3	0	3	13	40	53	112
FS4401	1	2	7	15	62	112	199
Jeju	3	1	5	17	67	131	224

Note: Start_nonsyn: non-synonymous mutations of start codon; Stop_nonsyn: non-synonymous mutation of termination codon; Premature_stop: nonsense mutation, the triplet codon mutates into a stop codon; Synonymous: synonymous mutation in the gene region; Nonsynonymous: non-synonymous mutations in the gene region; Intergenic：SNP in the intergenic region.

Table S7 The results of InDel annotation with 138A as the reference sequence

Sample ID	I-gene-middle	D-gene-middle	Intergenic	Total
138B	2	0	12	14
FS4401	6	13	45	64
Jeju	9	18	57	84

Note: I-gene-middle：insertion in CDS; D-gene-middle: deletion in CDS; Intergenic：Intergenic: InDel in the intergenic region.

Table S8 The type of genetic mutation caused by InDel with 138A as the reference sequence

Sample ID	Frame shifted	Other change	CDS with InDel
138B	0	2	2
FS4401	13	2	15
Jeju	21	4	25

Note: Frame shifted: InDel results in a frame-shift mutation of the open reading frame; Other Change: InDel has no significant effect on the open read frame; CDS with InDel: InDel in the gene region.

Table S9 The cytoplasmic genotypes of 43 materials and the identification result by CMS makers

Sample ID	cytoplasmic genotypes	Identification of orf300a	Identification of orf507, orf456, cox2 and acc D-U
18Q5025	normal	normal	normal
18Q5038	normal	normal	normal
18Q5043	normal	normal	normal
18Q5044	normal	normal	normal
18Q5048	normal	normal	normal
18Q5050	normal	normal	normal
18Q5055	normal	normal	normal
18Q5064	normal	normal	normal
18Q5109	normal	normal	normal
18Q5110	normal	normal	normal
18Q5111	normal	normal	normal
18Q5117	normal	normal	normal
18Q5120	normal	normal	normal
18Q5121	CMS	CMS	normal
18Q5132	normal	normal	normal
18Q5142	normal	normal	normal
18Q5144	normal	normal	normal
18Q5146	normal	normal	normal
18Q5169	normal	normal	normal
18Q5249	normal	normal	normal
18Q5250	normal	normal	normal
18Q5253	normal	normal	normal
18Q5268	normal	normal	normal
18Q5269	normal	normal	normal
18Q5270	normal	normal	normal
18Q5271	normal	normal	normal
18Q5273	CMS	CMS	normal
18Q5281	normal	normal	normal
18Q5423	CMS	CMS	normal
18Q5424	normal	normal	normal
18Q5425	CMS	CMS	normal
18Q5430	normal	normal	normal
18Q5439	normal	normal	normal
18Q5463	normal	normal	normal
18Q5464	normal	normal	normal
18Q5465	CMS	CMS	normal
18Q5466	normal	normal	normal
18Q5494	normal	normal	normal
18Q5496	CMS	CMS	normal
18Q5533	CMS	CMS	normal

18Q5562	normal	normal	normal
18Q5633	normal	normal	normal
18Q5634	normal	normal	normal

Table S10 Oligonucleotides used in this study

Primer names	Forward (5' – 3')	Reverse (5' – 3')	Reference	Application
orf456	ATGCCCAAAAGTCCCATGTA	TTACTCGGTTGCTCCATTGT	32	RT-PCR
orf507	ATGCCCAAAAGTCCCATGTA	TTAAAAAGCGCTAAACAAATTGC	33	RT-PCR
cox2	GATGCAGCGGAACCATGGCAATTA	ACTGCACTGACCATAGTAAACTCC	30	RT-PCR
control				SCAR-PCR
cox2 SCAR	GTCGGGAGAACTACCTAACTA	GGCTACCTAGTGATTTACAAGCA	30	SCAR-PCR
atp6 SCAR	AGTCCACTTGAACAATTTGAAATAATC	GTTCCGTACTTTACTTACGAGC	30	SCAR-PCR
acc D-U	GTGATTGGATTTCTATGCGG	GGAATGCCGTTTATTTGGCC	48	SCAR-PCR
orf292a	ATGAGACAAAGGAGAGCACTTAGACAATTC	TCACACGACAGGTGCACGATCTAC	In this study	RT-PCR
orf300a	ATGTTCTCACTCTTAAAAAAATTAAGAGC	TTAGGATTTCCAATTCCAATGC	In this study	RT-PCR
orf300a	AGAGCAGTTCAACGCAGAGCAG	CCGATCTTTTTGTCGCCCCTGA	In this study	real-time PCR
orf262a	ATGAAAAAATCGTTCCACTCGCAGC	TTACGCGGGTGAAGCTTGGG	In this study	RT-PCR
orf262a	TCTCCGTCGGACTCCCTTATCG	GCTCTTCGTTCTCCCCGTTGTT	In this study	real-time PCR
sdh3	ATGAATATCCTTCGCCCCTTATCTC	TCAATTTTGCACAGTCATTTCAATC	In this study	RT-PCR
rpl2	ATGAGACAAAGGAGAGCACTTAGAC	TTAGTCTGTATGGGCATTCTGG	In this study	RT-PCR
orf157a	CGGCCCTATTGTTCCGATGGAG	GGGCCGGAAGGACTTCTAGTCT	In this study	real-time PCR
orf115b	AAGAATTCGGCCTTTCAACGGGT	CCCATTCTGGGTCCGACTGTTTC	In this study	real-time PCR

Table S11 Sequence variations of mitochondrial genes coding for known and hypothetical proteins between 138A and 138B

Gene ID	position	Nucleotide variation (138B→138A)	Codon mutate	aa mutate
orf229a	31116	G→C	AGT→ACT	S→T
orf138a	96376	C→A	GCA→GAA	A→E
orf337a	148817	C→A	CAA→AAA	Q→K
orf675a	151284	G→A	GAT→AAT	D→N
orf249a	169197	C→G	CCA→GCA	P→A
	169315	T→C	TCT→TCC	S→S
mat-R	283488	A→G	GCA→GCG	A→A
orf104c	333230	C→G	ACA→AGA	T→R
	333235	T→G	ATT→ATG	I→M
orfB	311237-311698	AAACTGTTG		-NSL
orf25	162719-163306	CTGCATATT		-NMQ

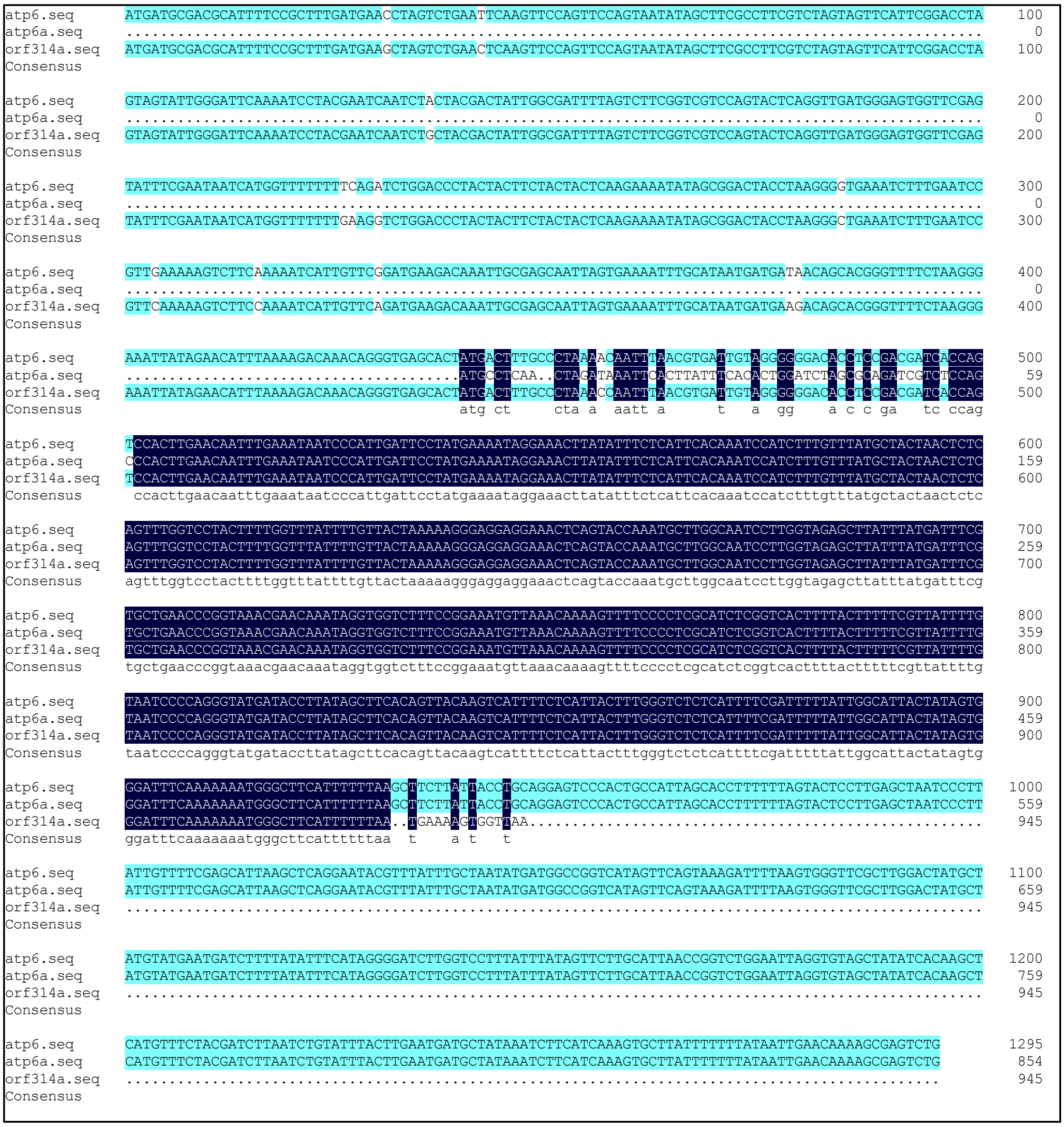

Figure S1 The sequences *atp6* in 138A and 138B

```
orf507.seq        ATGCCCAAAAGTCCCATGTATTTCTGGTTAAACAAACCAGCAATTTCCGACAAGTCTTTCTTCATTGGAAGAGCAAGAAGCGGAACTACAACATTTACAT   100
orf507-138A.seq   ATGCCCAAAAGTCCCATGTATTTCTGGTTAAACAAACCAGCAATTTCCGACAAGTCTTTCTTCATTGGAAGAGCAAGAAGCGGAACTACAACATTTACAT   100
orf507-138B.seq   ATGCCCAAAAGTCCCATGTATTTCTGGTTAAACAAACCAGCAATTTCCGACAAGTCTTTCTTCATTGGAAGAGCAAGAAGCGGAACTACAACATTTACAT   100
Consensus         atgcccaaaagtcccatgtatttctggttaaacaaaccagcaatttccgacaagtctttcttcattggaagagcaagaagcggaactacaacatttacat

orf507.seq        GCAATTTCACCATGAATTTTATTGATTATGGCACATTGTTTACTTTTTCTTTTTATCTCGGTATTTCAATCGGCATTTTTGCGGGCCGGTTTTTTGAGCG   200
orf507-138A.seq   GCAATTTCACCATGAATTTTATTGATTATGGCACATTGTTTACTTTTTCTTTTTATCTCGGTATTTCAATCGGCATTTTTGCGGGCCGGTTTTTTGAGCG   200
orf507-138B.seq   GCAATTTCACCATGAATTTTATTGATTATGGCACATTGTTTACTTTTTCTTTTTATCTCGGTATTTCAATCGGCATTTTTGCGGGCCGGTTTTTTGAGCG   200
Consensus         gcaatttcaccatgaattttattgattatggcacattgtttactttttctttttatctcggtatttcaatcggcatttttgcgggccggttttttgagcg

orf507.seq        AAGTGAAGTTTTACAGGAATTGGAGAACTTCCAGCTAGAAAAAAATAAAACTGAAAACGGAAGCAGAACTGCAATTTCTTTGTAGAGAGCACTTGAGAATG   300
orf507-138A.seq   AAGTGAAGTTTTACAGGAATTGGAGAACTTCCAGCTAGAAAAAAATAAAACTGAAAACGGAAGCAGAACTGCAATTTCTTTGTAGAGAGCACTTGAGAATG   300
orf507-138B.seq   AAGTGAAGTTTTACAGGAATTGGAGAACTTCCAGCTAGAAAAAAATAAAACTGAAAACGGAAGCAGAACTGCAATTTCTTTGTAGAGAGCACTTGAGAATG   300
Consensus         aagtgaagttttacaggaattggagaacttccagctagaaaaaaataaaactgaaaacggaagcagaactgcaatttctttgtagagagcacttgagaatg

orf507.seq        AATGAAGAATTACAATTACCTGTTCCAGATGGAACGAGTATGCACATCTCCGACTTTTTAGGGAAAGCCTTTTTGGTCGACGAGACTGTGAGGGAACGAA   400
orf507-138A.seq   AATGAAGAATTACAATTACCTGTTCCAGATGGAACGAGTATGCACATCTCCGACTTTTTAGGGAAAGCCTTTTTGGTCGACGAGACTGTGAGGGAACGAA   400
orf507-138B.seq   AATGAAGAATTACAATTACCTGTTCCAGATGGAACGAGTATGCACATCTCCGACTTTTTAGGGAAAGCCTTTTTGGTCGACGAGACTGTGAGGGAACGAA   400
Consensus         aatgaagaattacaattacctgttccagatggaacgagtatgcacatctccgactttttagggaaagcctttttggtcgacgagactgtgagggaacgaa

orf507.seq        TATTAGGGCTGACTCAAATTTATATGGATCTAAAAAACAATGGAGCAACGAGTAACTTTTTTCTTTTATTTTTAGACTATTATAGCAATTTGTTTAGCGC   500
orf507-138A.seq   TATTAGGGCTGACTCAAATTTATATGGATCTAAAAAACAATGGAGCAACGAGTAACTTTTTTCTTTTATTTTTAGACTATTATAGCAATTTGTTTAGCGC   500
orf507-138B.seq   TATTAGGGCTGACTCAAATTTATATGGATCTAAAAAACAATGGAGCAACGAGTAACTTTTTTCTTTTATTTTTAGACTATTATAGCAATTTGTTTAGCGC   500
Consensus         tattagggctgactcaaatttatatggatctaaaaaacaatggagcaacgagtaacttttttcttttatttttagactattatagcaatttgtttagcgc

orf507.seq        TTTTTA   506
orf507-138A.seq   TTTTTA   506
orf507-138B.seq   TTTTTA   506
Consensus         ttttta
```

Figure S2 The sequences *orf507* in 138A and 138B
